# Varying redox potential affects P release from stream bank sediments

**DOI:** 10.1371/journal.pone.0209208

**Published:** 2018-12-14

**Authors:** Suroso Rahutomo, John L. Kovar, Michael L. Thompson

**Affiliations:** 1 Indonesian Oil Palm Research Institute, Medan, North Sumatra, Indonesia; 2 National Laboratory for Agriculture and the Environment, USDA-Agricultural Research Service, Ames, IA, United States of America; 3 Agronomy Department, Iowa State University, Ames, IA, United States of America; University of Delaware, UNITED STATES

## Abstract

Sediments in streams that drain agricultural watersheds may be sinks that can adsorb P from the stream or sources that can release P to the stream. Sediment characteristics and environmental factors, including the oxidation-reduction (redox) potential of the water associated with the sediment, determine whether P will be adsorbed or released by the sediment. We investigated P adsorption and release by four sediments [three Holocene-age sediments (Camp Creek, Roberts Creek and Gunder) as well as Pre-Illinoian-age Till] that occur in Walnut Creek, a second-order stream in Jasper County, Iowa, that is representative of many small streams in the glaciated upper Midwest of the US. The effects of two redox potentials on phosphorus buffering capacity (PBC) and equilibrium phosphorus concentration (EPC) were evaluated in batch adsorption experiments. We also simulated aerobic and anerobic conditions over a 24-day period and measured solution-phase P concentrations in stirred systems where the sediments were isolated from the water by dialysis tubing. The batch experiment indicated that the EPCs of the three Holocene-age sediments were similar to one another and increased with decreasing redox potential. In the stirred flow reactors, more dissolved P was released from the Camp Creek and Roberts Creek sediments under anaerobic conditions than from the other sediments. This observation suggests that these two sediments, which are younger and higher in the stratigraphic sequence, are more likely to be P sources in suboxic settings. The P buffering capacity was greatest in the till. Where it is in contact with the stream water, the till is likely to serve as an adsorbing sink for P in the water column.

## Introduction

Phosphorus export from agricultural regions contributes to stream eutrophication at both local and regional scales [1, 2]. Soil materials in floodplain sediments and those eroded in runoff from agricultural land contribute to elevated P levels in stream waters. Once deposited with sediment in a water body, the solid-phase P may function as a source to the overlying water column [3, 4]. When nitrogen is not limiting for phytoplankton growth in a stream, even a small increase in P may lead to eutrophication. This reflects the small stoichiometric ratio in biomass for P compared to other major nutrients necessary for algal growth. The classic example is 106C:16N:1P atomic ratio for phytoplankton in marine environments [5].

In contrast to their function as sources of P, sediments may also act as P sinks when in contact with stream water along streambanks, in the stream bottom, or as suspended particles. Phosphate ions are readily adsorbed by Fe, Al, or Mn oxides in many sediments, although other adsorption sites associated with layer silicates, oxide—organic matter complexes, or Ca and Mg carbonates in calcareous soil materials, may also be present [6–8]. Because the oxidation state of Fe and Mn can vary with oxidation-reduction (redox) potential, the stability of Fe and Mn oxides varies in aerobic and anaerobic environments, influencing the P dynamics of sediments [9].

Reducing conditions have an impact on pH and dissolved organic matter in soils and sediments [10]. Reduction of metal ions, such as Fe, consumes protons, raising the pH of the solution. Reduction of Fe leads to destabilization of poorly crystalline Fe oxides such as ferrihydrite that can release adsorbed or occluded P as they break up. The rise in pH also results in deprotonation of both organic matter functional groups and the surfaces of Fe or Mn oxides, leading to increases in dissolved organic carbon (DOC) and negatively charged surfaces [11]. Desorption of P from minerals has been attributed to competition for adsorption sites between phosphate ions and low-molecular-mass organic ions on sesquioxides [12] or to organic complexation of metal ions that leads to dissolution of phosphate-bearing solid phases [13]. There is still uncertainty about the concentrations of dissolved organic matter that would be required to significantly inhibit P adsorption in soils [14]. In the context of anaerobic stream sediments, it may be hypothesized that redox and pH-induced increases in DOC can lead to increases in dissolved P.

In a freshwater stream, aerobic environments are likely where flowing water promotes the dissolution of atmospheric oxygen. On the other hand, when water moves slowly or becomes stagnant (e.g., blocked by debris dams or beaver dams), dissolved oxygen enters the water more slowly [15,16]. Further, anaerobic environments are promoted by the decomposition of sedimentary organic matter and aquatic microbial biomass. In addition, anaerobic environments may occur in the hyporheic zone where anaerobic groundwater is discharged into stream sediments [17].

The Walnut Creek watershed is a 5218-ha, 12-digit HUC watershed in Jasper County, Iowa, with a variety of land uses typical of the region, including row-crop production, pasture, and riparian forest. The stream is representative of many in the glaciated upper Midwest of the US that have incised sediments affected by recent agricultural practices as well as older Holocene sediments. The fate of P in the stream and its watershed have been extensively studied with respect to channel storage, stream bank erosion, and suspended sediment transport [18, 19] as well as riparian groundwater dynamics [20, 21, 22, 23, 24]. In addition, we have recently characterized the amounts and distribution of the forms of P in bank sediments, in-stream sediments, and floodplain sediments of Walnut Creek [25], and we have identified the forms of P in the sediments that are most susceptible to release to the water column under low redox conditions [26].

During much of the year, flow in Walnut Creek is derived mainly from low-redox-potential groundwater discharge that enters the channel through the Gunder Member of the Holocene DeForest Formation [22, 24]. The groundwater carries low concentrations of P (averaging about 0.05 mg L^-1^). During periods of high flow, two overlying, younger sediments, the Roberts Creek and Camp Creek Members, as well as (in some parts of the watershed) incised pre-Illinoian Till, become water-saturated near the stream channel during such high-discharge events, even as groundwater continues to discharge into the stream [20]. While flooding of the sediments in and adjacent to the channel does not last for weeks, saturated pores (anaerobic microsites) are likely to persist for long periods in these sediments.

The primary objective of this study was to investigate P adsorption by and release from Walnut Creek stream bank sediments at varying redox potentials. In the first of two experiments, an adsorption study was conducted to investigate the effects of varying redox potential on phosphorus buffering capacity (PBC) and equilibrium phosphorus concentration (EPC), two useful sorption indices for evaluating whether sediments act as sinks or sources of P [27, 28, 29]. The second experiment was conducted to evaluate P release from sediments to the water under oxic, suboxic, and anoxic conditions. While the adsorption experiment measured sorption characteristics of the sediments under equilibrium conditions that might occur when sediment is resuspended during stream flow [20], the second experiment attempted to simulate P dynamics when groundwater with low oxygen concentrations enters the stream by passing through sediments [30]. In both experiments, we hypothesized that the concentrations of P retained by or released from the stream sediments to the aqueous phase would increase as the redox potential of the sediment–water systems declined.

## Materials and methods

### Sediment sampling and characterization

**Fig 1:**
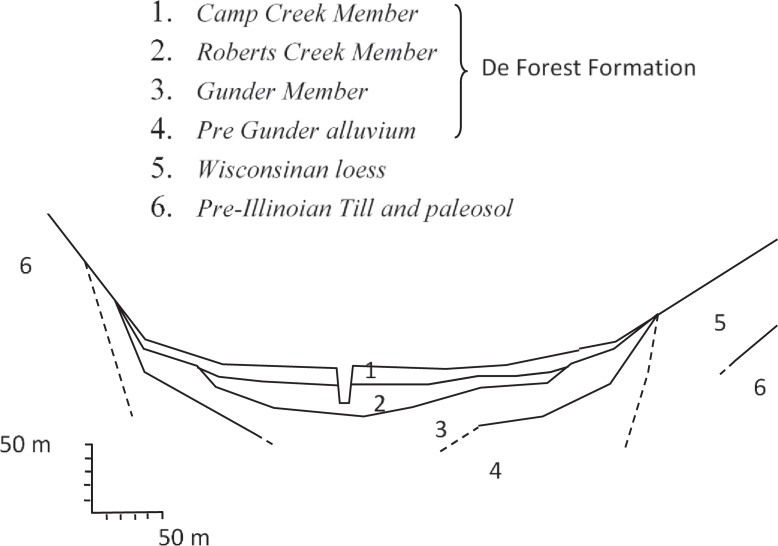
A schematic alluvial cross section through alluvium in the Walnut Creek watershed, adapted from Schilling et al. [20]. In some locations, the channel is close enough to the valley wall that the stream contacts Pre-Illinoian Till.

Walnut Creek is a second-order stream in Jasper County, Iowa. Samples of sediment from the watershed were collected to represent the major alluvial units in the Walnut Creek floodplain: the Camp Creek Member, the Roberts Creek Member, the Gunder Member (all members of the Holocene-age DeForest Formation), and Pre-Illinoian Till (Fig 1). The coordinates of the sampling site are 41° 33.382' N and 93° 15.887' W. Immediately after transport to the laboratory, a portion of each sample was subsampled and stored at 4°C, while another portion was air dried and sieved to pass a 2-mm screen. The samples stored in the cold room were prepared for the adsorption and desorption studies, and the <2-mm air-dried samples were used for sediment characterization.

Total nitrogen was determined by high-temperature dry combustion [31]. Total organic matter was determined using a loss-on-ignition method [32]. Particle size distribution was determined gravimetrically [33], while pH was determined potentiometrically at a soil:water ratio of 1:1. Perchloric acid digestion was used to determine total P [34]. Phosphorus concentrations in the digests were determined with the molybdate blue-ascorbic acid colorimetric method [35]. Mehlich-3 solution [36] was used to extract exchangeable cations (Ca_M3_, Mg_M3_, K_M3_, Na_M3_), and their concentrations were determined using inductively coupled plasma-atomic emission spectrometry. Mehlich-3 solution was also used to extract P (P_M3_) and the concentrations were determined using the ascorbic acid-molybdate blue method [35]. Citrate-bicarbonate-dithionite-extractable Fe (Fe_CBD_) was determined by extraction and atomic absorption spectroscopy [37]. Ammonium-oxalate-extractable Fe, Al, and Mn (Fe_ox_, Al_ox_, Mn_ox_) were determined by ICP-AES, while P (P_ox_) in this extraction was determined colorimetrically using the malachite green method [38].

### Phosphorus adsorption experiment

A batch adsorption experiment was used to assess the amount of P adsorbed by the stream sediments (adsorbed solid-phase P, Q) as a function of phosphate concentration in the solution (liquid-phase P, C) after equilibration at a constant temperature. As a background solution, stream water was used to maintain ionic strength at a natural level [28]. Stream water collected from Walnut Creek was filtered through a 0.45-μm cellulose-acetate membrane filter. The water had a pH of 8.0 (±0.1), electrical conductivity (EC) of 0.45 (±0.04) dS m^-1^, and dissolved P of 0.050 (± 0.009) mg L^-1^. Phosphorus was added to separate volumes of the filtered stream water to attain initial P concentrations of 0.05, 0.10, 0.15, 0.25, 0.45, and 0.85 mg P L^-1^.

The experiment was set up as a completely randomized design involving three treatments (without anaerobic incubation (A), with anaerobic incubation (AN), and with anaerobic incubation with addition of glucose (ANG)). Each treatment was replicated three times, with 30 mL of P-spiked base solution added to a 50-mL centrifuge tube containing moist sediment equivalent to 1.5 g of oven-dried weight. For the AN treatment, the cap of the centrifuge tube was lined with a rubber septum to allow purging each sample with nitrogen (N_2_). Nitrogen purging was conducted at the beginning of the incubation and repeated weekly. To minimize air infiltration, silicone glue was applied to the surface of the rubber septum and bottom part of the cap after the N_2_ purge. The AN treatment samples were incubated for 30 days in a chamber filled with N_2_. A similar anaerobic incubation process was used for the ANG treatment, except that glucose (C_6_H_12_O_6_, Sigma) was dissolved in the P-spiked base solutions to attain a concentration of 0.25 g glucose L^-1^. This high concentration of glucose (~100 mg C L^-1^) was chosen to promote microbial respiration and to ensure low redox conditions in the treatment.

**Fig 2:**
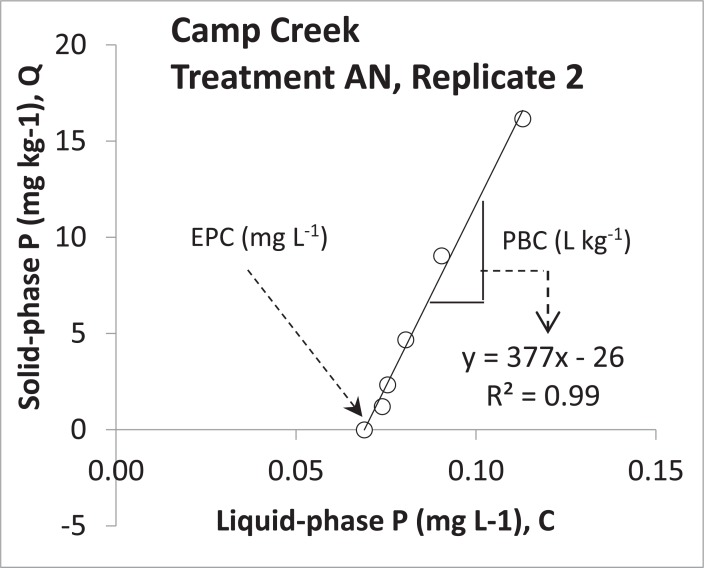
To determine phosphorus buffering capacity (PBC) and equilibrium phosphorus concentration (EPC) in the isotherm adsorption experiment the liquid phase P (C) was plotted against P in the solid phase (Q). This relationship is exemplified by one of the anaerobic treatments of Camp Creek sediment.

Treatment A used amounts of sediment sample and P-spiked base solution similar to those of the incubation treatments, however the sediments were shaken at 200 excursions per minute for 24 hours, not incubated for 30 days. After the sediments settled, Eh and pH were measured by inserting probes into the tube so that the edge of the probe was about 2 cm above the surface of the settled sediment. Eh and pH were measured only for the spiked levels of 0.10 and 0.20 mg P L^-1^. For the AN and ANG treatments, Eh and pH were measured in a glove box which was filled with N_2_. Afterward, each tube from all treatments was centrifuged at 4,300 x *g* for 15 minutes, followed by filtering the supernatant through a 0.45-μm cellulose-acetate membrane filter. Equilibrium phosphate concentration in the solution was determined using a malachite green colorimetric method [38]. The equilibrium phosphorus concentration (EPC) and the phosphorus buffering capacity (PBC) were determined following the method described by Hongthanat *et al*. [28] in which P in the liquid phase (C) was plotted against P in the solid phase (Q), and a line was fit to the data using simple linear regression. An example for determining PBC and EPC of the Camp Creek sediment is shown in Fig 2.

### Quantifying P release from the sediments

**Fig 3:**
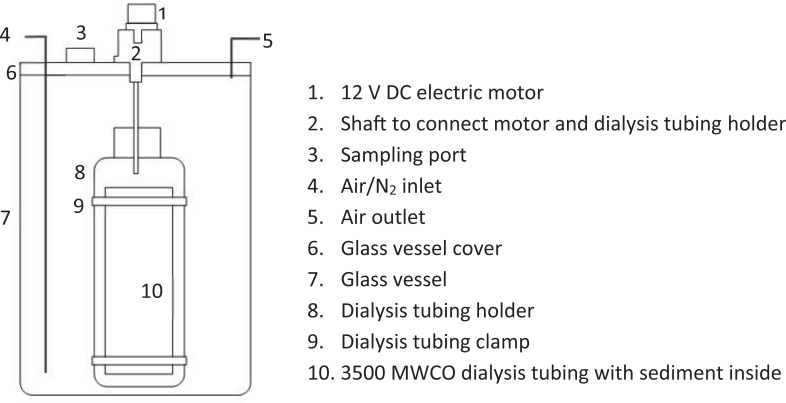
Schematic design of reactor for quantifying P release from the sediments under oxic and anoxic environments.

While P adsorption (retention) was investigated with static batch equilibrium experiments, P release from the sediments under oxic and anoxic conditions was quantified by using a stirred reactor specifically designed for this study (Fig 3). The main vessel of the reactor was a glass canning jar with the lid modified to attach a small electric motor, inlet and outlet gas tubing, dialysis tubing holder, and an access port. The electric motor was powered by 12 V DC power source, connected to a shaft that rotated the dialysis tubing holder at 12 rpm for 8 hours per day. Since a metal may function as an electron acceptor and promote Fe^3+^ reduction in the sediments [39], the shaft and the dialysis tubing holder were constructed of plastic parts. The dialysis tubing (Fisher Scientific) had a 3,500 Da molecular weight cut off (~2 nm pore diameter). In the aerobic treatment, an oxidizing environment in the reactor was maintained by continuously pumping air through the inlet tubing, while air from inside the reactor was expelled through the outlet tubing. A reducing environment was produced by a continuous N_2_ purge through the inlet tubing. The access port was designed to fit a 10-mL pipet, pH probe, Eh probe, and thermometer; it could be opened easily and closed tightly after opening.

The experimental design was a split-plot, in which sediment type and gas purging treatment were assigned as main plot and subplot, respectively. Four sediments (Camp Creek, Roberts Creek, Gunder, and Pre-Illinoian Till), two gas purging treatments (air and N_2_ purging), and three replications were used in the experiment, requiring 24 reactors. To arrange the reactors following the split-plot design, a lab bench was divided into three blocks. Each block was divided into four plots, and each plot was randomly assigned to one of the four sediment types. Then each plot was divided into two subplots; one subplot was randomly assigned to a reactor purged with air and another subplot was assigned to a reactor purged with N_2_.

To simulate common stream characteristics in Iowa, the matrix solution used in this experiment was prepared with deionized water containing 1.3 *mM* CaCl_2_, 0.1 *mM* CaCO_3_, and 0.4 *mM* MgCl_2_•6H_2_O. The electrical conductivity of the base solution was 0.45 dS m^-1^ and the pH was 8.0; DOC and orthophosphate concentrations were below analytical detection limits (0.006 mg L^-1^). To maintain the solid:solution ratio of the batch experiment (1:20), the initial volume of the base solution in each reactor was 600 mL, and the mass of moist sediment inside the dialysis tubing was equivalent to 30 g of oven-dried sediment. Solution samples were collected from each reactor with a 10-mL volumetric pipette on days 3, 6, 9, 12, and 24. Dissolved P and DOC were determined immediately after samples were collected. The P concentration was determined using the malachite green colorimetric method, and DOC was measured by high-temperature combustion using a Shimadzu TOC 5050 (Shimadzu Corp.). Redox potential (Eh), pH, and temperature were also measured at each sampling time.

Statistical analyses were performed with SAS version 9.4 [40]. These analyses included simple regression to fit linear adsorption models, analyses of variance to compare the effects of the incubation treatments on adsorption parameters as well as on Eh and pH, and paired *t*-tests to compare the impacts of aerobic and anaerobic conditions on Eh, pH, DOC, and dissolved P in equilibrating solutions in the P release experiment.

## Results and discussion

### Sediment properties

Selected chemical and physical properties of sediments measured in this study are summarized in Table 1. These samples were chosen to represent the properties of a larger set of Walnut Creek sediment samples that were described by Rahutomo et al. [25]. There was a fairly narrow range of clay contents across the sediment types, while the highest sand content was found in the Pre-Illinoian Till. The pH values of the Camp Creek and Roberts Creek sediments were slightly acidic (6.2 and 6.3, respectively), while those of the Gunder sediment and the till were alkaline (7.4 and 8.1, respectively). The till was calcareous. The lowest and highest concentrations of dithionite-extractable Fe were found in the Gunder sediments and the till, respectively (Table 1). In the Walnut Creek channel, the Gunder sediments normally occur below the water table, and perhaps concomitant low redox potentials have limited mineral weathering and the precipitation of secondary Fe oxides in these materials. The relatively high level of Fe oxides in the much older till was also reflected by its yellowish brown color. The youngest sediments, Camp Creek and Roberts Creek, had greater organic matter, total N, and oxalate-extractable Fe and Al concentrations than the Gunder sediments and the till. The range of TP, P_M3_, and P_ox_ was 473 to 588, 5 to 41, and 106 to 193 mg kg^-1^, respectively. In all these ranges, the lowest and highest values were in the till and Roberts Creek sediments, respectively.

**Table 1:** Selected sediment properties, where OM = organic matter, TP = total P, Fe_ox_/Al_ox_/Mn_ox_/P_ox_ = ammonium oxalate extractable Fe/Al/Mn/P, Fe_CBD_ = citrate bicarbonate dithionite extractable Fe, Ca_M3_/Mg_M3_/K_M3_/Na_M3_/P_M3_ = Mehlich-3 extractable Ca/Mg/K/Na/P.

Sediment properties	Camp Creek	Roberts Creek	Gunder	Pre-Illinoian Till
**pH (H**_**2**_**O, 1:1)**	6.2	6.3	7.4	8.1
**Sand (%)**	11	13	6	49
**Silt (%)**	64	60	72	30
**Clay (%)**	25	27	22	21
**OM (%)**	3.38	4.51	1.51	1.71
**Total N (%)**	0.14	0.14	0.06	0.03
**Fe**_**CBD**_ **(mg kg**^**-1**^**)**	6,825	5,862	3,813	10,124
**Fe**_**ox**_ **(mg kg**^**-1**^**)**	3,348	3,947	1,967	743
**Al**_**ox**_ **(mg kg**^**-1**^**)**	1,002	1,076	445	235
**Mn**_**ox**_ **(mg kg**^**-1**^**)**	702	1,263	154	166
**P**_**ox**_ **(mg kg**^**-1**^**)**	158	193	137	106
**Ca**_**M3**_ **(cmol (+) kg**^**-1**^**)**	9.88	14.92	10.68	33.75
**Mg**_**M3**_ **(cmol (+) kg**^**-1**^**)**	3.86	4.65	4.22	2.40
**K**_**M3**_ **(cmol (+) kg**^**-1**^**)**	0.26	0.37	0.30	0.26
**Na**_**M3**_ **(cmol (+) kg**^**-1**^**)**	0.30	0.39	0.24	0.25
**P**_**M3**_ **(mg kg**^**-1**^**)**	29	41	28	5
**TP (mg kg**^**-1**^**)**	491	588	484	473

### Adsorption experiment

**Fig 4:**
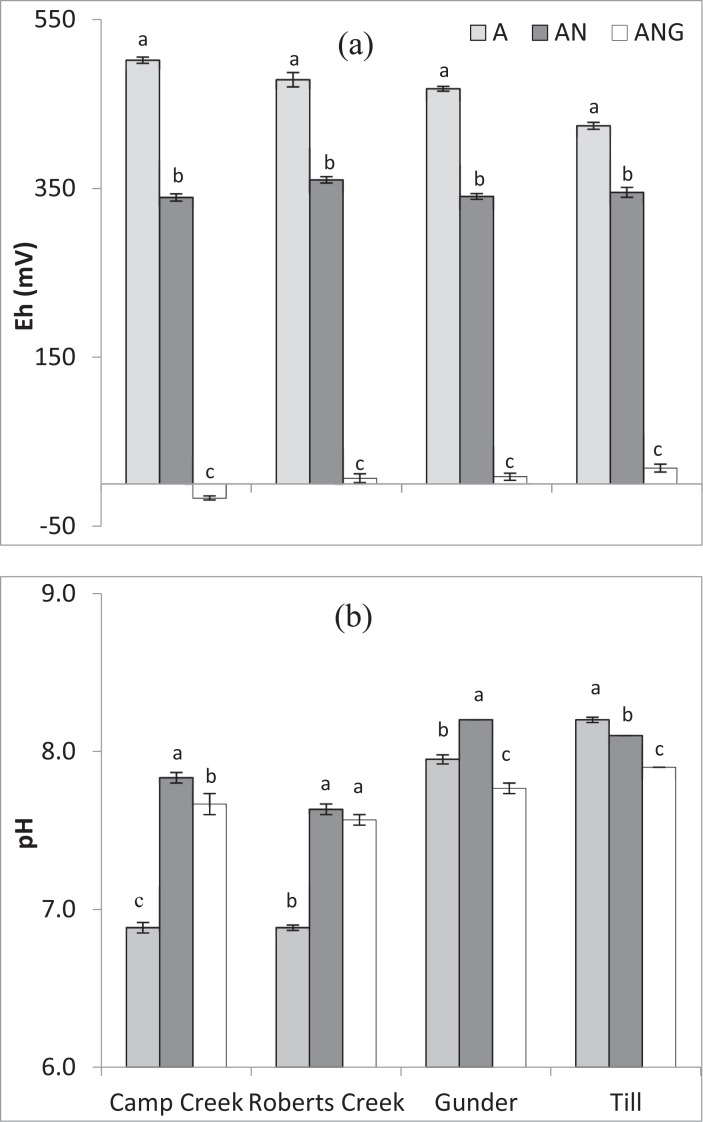
Effects of the treatments (A = without anaerobic incubation, AN = with anaerobic incubation, and ANG = with anaerobic incubation + glucose) on redox potential (Eh) (a) and pH (b) at 24 hours for Treatment A and at 30 days for Treatments AN and ANG. Means with different letters within the same sediment are significantly different (least significant difference test at α = 0.05). Error bars represent standard error.

We simulated low redox potential that might be experienced by these sediments by using a laboratory incubation experiment. For all sediment samples, anaerobic incubation lowered redox potential of the solution at equilibrium. When glucose was present in the base solution, a further decrease in redox potential was measured (Fig 4). Across the four sediment samples, the mean Eh values under AN (anaerobic incubation) and ANG (anaerobic incubation + glucose) treatments ranged from 339 to 360 mV and -17 to 19 mV, respectively. Several observations may be considered regarding these Eh values. First, the Eh values after 30 days of anaerobic incubation without addition of glucose were somewhat greater than 300 mV and corresponded to suboxic conditions [41]. Second, the mean Eh values in the ANG treatment were significantly lower than those of the AN treatment, indicating the effect of a readily bioavailable carbon source, i.e., glucose, in promoting a more anaerobic, i.e., anoxic environment. Third, the Eh values we report were measured approximately 2 cm above the settled sediment. The Eh values may have differed if the electrode had been closer to the settled sediment or inserted into the settled sediment. For example, Hill and Robinson [42] showed a sharp decrease in Eh at the sediment-water interface compared to that of an overlying water column. Redox measurements in sediment-water systems are often challenging to interpret because of irreversible reduction reactions involving oxygen and nitrogen gases and because of the presence of multiple, simultaneous redox couples [41]. Overall, the Eh values that we report here should be compared to one another and not directly to those of other studies or thermodynamically derived reference values.

The decrease of redox potential under the anaerobic incubation in this study was likely the result of several processes. During the early stage of anaerobic incubation, oxygen was depleted as aerobic microorganisms utilized available oxygen as an electron acceptor. In the absence of oxygen, microorganisms adapted to anaerobic conditions became more competitive due to their ability to use nitrate, Mn^4+^, and Fe^3+^ as electron acceptors [43], further decreasing the redox potential. Finally, oxygen depletion was probably accelerated when N_2_ was periodically introduced to the reaction vessel. The first and second mechanisms were likely enhanced by the abundance of glucose as an energy source and electron donor in the ANG treatment, resulting in a dramatic drop in the redox potential.

The response of equilibrium pH to the anaerobic incubation treatments differed among the four sediments (Fig 4). For the Camp Creek, Roberts Creek, and Gunder sediments, the pH values were significantly higher after anaerobic incubation than without anaerobic incubation. In contrast, the pH of the AN treatment of the till was slightly lower than the pH of the till without anaerobic incubation. For the Camp Creek sediment, the Gunder sediment, and the till sediments, addition of glucose to the anaerobic incubation resulted in significantly lower pH than in the AN treatment with no glucose. In the Roberts Creek sediment, the addition of glucose did not change pH of the anaerobic treatments (Fig 4). Protons are consumed in reduction processes [41], raising the pH of non-alkaline soils. However, alkaline soils are usually buffered sufficiently to prevent significant changes in pH as reduction proceeds [43].

The EPC values of the aerobic, non-incubated sediments (ranging from ~0.01 to ~0.04 mg L^-1^) were roughly similar to those reported by Hongthanat et al. [28] and Hongthanat et al. [44] for sediments in several streams of the Rathbun Lake watershed in southern Iowa (which ranged from 0.01 to 0.23 mg L^-1^) (Table 2). The PBC values of the sediments without anaerobic incubation in the present study, averaging 404 L kg^-1^, were somewhat lower than those of the stream sediments reported by Hongthanat et al. [44], which averaged 511 L kg^-1^.

**Table 2:** Effects of varying redox potential on phosphorus buffering capacity (PBC, in L kg^-1^) and equilibrium phosphorus concentration (EPC, in mg L^-1^).

Treatments	Camp Creek		Roberts Creek		Gunder		Pre-Illinoian Till	
	PBC	EPC	PBC	EPC	PBC	EPC	PBC	EPC
**A**	188 b	0.036 b	326 a	0.030 b	132 b	0.029 c	970 a	0.011 b
**AN**	387 a	0.063 b	522 a	0.047 b	179 a	0.085 b	173 b	0.011 b
**ANG**	35 c	0.835 a	45 b	0.993 a	16 c	1.313 a	200 b	0.018 a

Treatment A is without anaerobic incubation, AN is with anaerobic incubation, ANG is with anaerobic incubation and addition of glucose. Means with the same letter within the same column are not significantly different (least significant difference at α = 0.05).

**Fig 5:**
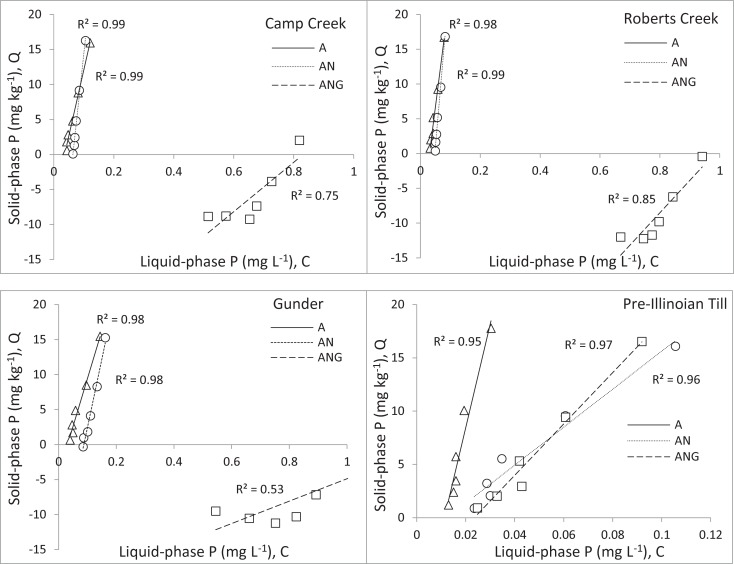
Phosphorus adsorption isotherms developed with simple linear regression for four sediments subjected to three experimental treatments (A = without anaerobic incubation (Δ), AN = with anaerobic incubation (○), and ANG = with anaerobic incubation + glucose (□). Each of the C and Q values plotted in the graph represents the mean of three replicates.

In comparison to treatment A (without anaerobic incubation), anaerobic incubation (treatment AN) significantly increased the PBC values of the Camp Creek and Gunder sediments, while in the Roberts Creek sediments, there was no change in PBC (Table 2, Fig 5). Unlike the other sediments, the PBC values of the till dropped significantly from 970 L kg^-1^ (without anaerobic incubation) to 173 L kg^-1^ (with anaerobic incubation). Only the Gunder sediments showed a significant increase in EPC when samples were equilibrated under anaerobic conditions. When glucose was added to the anaerobic incubation (treatment ANG), all sediments had similar responses: the PBC values were significantly lower than those without anaerobic incubation. The lower PBC values in the ANG treatment coincided with significantly higher EPC values than those in other treatments.

An increase in EPC values due to anaerobic incubation has been reported in previous studies [9, 45, 46, 47]. Moreover, Pant and Reddy [9] showed that anaerobic incubation also resulted in a decrease in the maximum P adsorption capacity. In the present study, the increase in EPC and decrease in PBC were exhibited by all four sediments when treated with anaerobic incubation + glucose. It is likely that the deeply anoxic environment created with an abundant energy source promoted reduction of Fe^3+^ to Fe^2+^ and dissolution of Fe oxides, resulting in fewer sorption sites and the release of Fe-bound P. This was consistent with the significant drop of PBC values, which reflected a decrease in the capability of the solid phase to buffer increasing levels of P in the liquid phase.

As noted earlier, anaerobic incubation (without addition of glucose) led to higher PBC values than when no anaerobic incubation was applied. This result may be compared to the reports of Reddy *et al*. [45] and Nair *et al*. [46], who demonstrated that the maximum P adsorption capacity increased with anaerobic incubation. Brand-Klibanski *et al*. [47] suggested that the higher adsorption capacity of reduced soil could be linked to precipitation of mineral P, particularly at higher P concentrations. In the present study, precipitation of mineral P under anaerobic conditions may have contributed to buffering the elevated level of liquid phase-P, especially at the higher base solution P levels, reflected by higher PBC values.

Nevertheless, a different process must have occurred in the till, where PBC significantly decreased with anaerobic incubation, even without addition of glucose. The till contained less ammonium oxalate-extractable Fe, but more dithionite-extractable Fe and more Mehlich-3-extractable Ca than the other sediments (Table 1). The higher PBC values and lower EPC values in the till (compared with the other sediments) could be attributed to P adsorption by Fe oxides as well as to precipitation of Ca-P minerals, even under the water-saturated and reducing conditions of the suboxic and anoxic treatments.

### P release experiment

**Fig 6:**
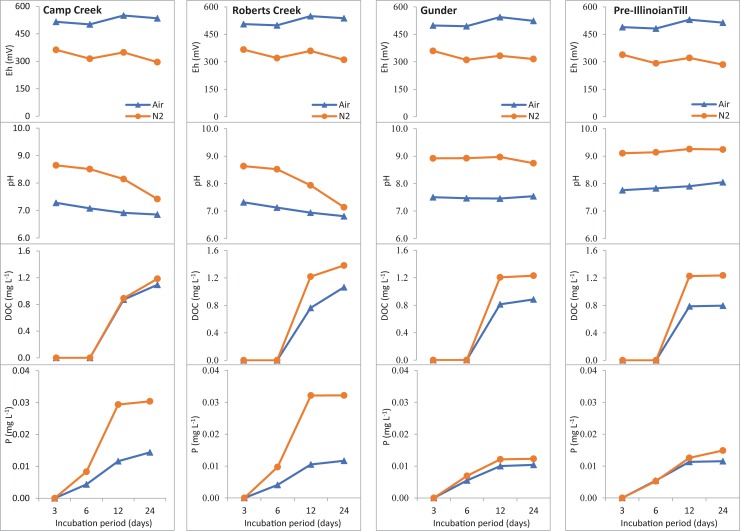
Dissolved P, dissolved organic carbon (DOC), pH, and redox potential (Eh) under aerobic (air purging, -Δ-) and anaerobic (N_2_ purging, -○-) environments in the solution for each sediment during the 24 days of incubation. Each point represents the mean of three replicates.

During the P release experiment using stirred reactors, we monitored redox potential, pH, dissolved organic carbon, and dissolved P concentrations in the solution for each sediment on days 3, 6, 12, and 24 of the incubation (Fig 6). The differences in these parameters between the aerobic and anaerobic (air and N_2_ purge) treatments are summarized in Table 3.

**Table 3:** Differences between aerobic conditions (with air purging) and anaerobic conditions (with nitrogen purging) in redox potential (Eh), pH, dissolved organic carbon (DOC), and dissolved P in the solution for each sediment during 24 days of incubation. DOC and P concentrations are in mg L^-1^, and Eh is in mV. Compare with Fig 6.

Parameters/ sediment types	Day 3		Day 6		Day 12		Day 24	
	Diff.^a^	*p*^b^	Diff.^a^	*p*^b^	Diff.^a^	*p*^b^	Diff.^a^	*p*^b^
***Redox potential (Eh*, *mV)***								
**Camp Creek**	153	<0.0001	188	<0.0001	202	<0.0001	240	<0.0001
**Roberts Creek**	140	<0.0001	179	<0.0001	190	<0.0001	227	<0.0001
**Gunder**	139	<0.0001	184	<0.0001	211	<0.0001	208	<0.0001
**Pre-Illinoian Till**	151	<0.0001	190	<0.0001	209	<0.0001	230	<0.0001
***pH***								
**Camp Creek**	-1.37	<0.0001	-1.43	<0.0001	-1.23	<0.0001	-0.57	<0.0001
**Roberts Creek**	-1.32	<0.0001	-1.40	<0.0001	-1.00	<0.0001	-0.33	0.0009
**Gunder**	-1.42	<0.0001	-1.46	<0.0001	-1.51	<0.0001	-1.20	<0.0001
**Pre-Illinoian Till**	-1.35	<0.0001	-1.31	<0.0001	-1.36	<0.0001	-1.19	<0.0001
***Dissolved organic carbon (DOC*, *mg L***^***-1***^***)***								
**Camp Creek**	*n*.*d*.^c^	*n*.*d*.	*n*.*d*.	*n*.*d*.	0.03	0.63	- 0.07	0.53
**Roberts Creek**	*n*.*d*.	*n*.*d*.	*n*.*d*.	*n*.*d*.	- 0.43	0.0002	- 0.33	0.01
**Gunder**	*n*.*d*.	*n*.*d*.	*n*.*d*.	*n*.*d*.	- 0.43	0.0002	- 0.33	0.01
**Pre-Illinoian Till**	*n*.*d*.	*n*.*d*.	*n*.*d*.	*n*.*d*.	- 0.43	0.0002	- 0.47	<0.002
***Dissolved P (mg L***^***-1***^***)***								
**Camp Creek**	*n*.*d*.	*n*.*d*.	-0.004	0.05	-0.018	<0.0001	-0.016	<0.0001
**Roberts Creek**	*n*.*d*.	*n*.*d*.	-0.005	<0.02	-0.022	<0.0001	-0.021	<0.0001
**Gunder**	*n*.*d*.	*n*.*d*.	-0.001	0.47	-0.002	0.06	-0.002	0.39
**Pre-Illinoian Till**	*n*.*d*.	*n*.*d*.	0.000	0.85	-0.001	0.18	-0.004	0.13

^a^ Difference of least squares means at α = 0.05 (air purging–N_2_ purging treatments)

^b^ p- value

^c^ n. d. = not detected.

In the four sediment types, the N_2_ purge during days 3 to 24 resulted in significantly lower Eh (~ 300 mV) compared to the ambient air purge (~ 500 mV). As noted earlier, an Eh of 300 mV can be considered suboxic conditions [41]. On that basis, we assumed that a suboxic condition had developed in the reactors purged with N_2_. On the other hand, oxic conditions were likely maintained in the reactors that were continuously purged with air.

In contrast to Eh, anoxic conditions consistently resulted in higher pH values in the water than did oxic conditions throughout the incubation period (Fig 6). This result was in line with a previous study in a controlled experiment by Jiang *et al*. [48], who found that water overlying sediment under anoxic conditions had higher pH values than that under oxic conditions. Interestingly, there was a different pattern of pH changes under oxic and anoxic environment among the sediment types. The pH under oxic conditions remained stable at ~7.5 in the Gunder system and ~7.9 in the till system, and both increased to ~9.0 under anoxic conditions. In the Camp Creek and Roberts Creek systems, the pH under oxic conditions slightly decreased, from 7.3 on day 3 to 6.9 on day 24. Under anoxic conditions, the water pH values were both ~8.6 on day 3; then on day 24 the pH decreased to 7.4 and 7.1 in the Camp Creek and Roberts Creek systems, respectively.

Some variation in the pH of the solutions might be attributed to the gas purges. The industrial grade N_2_ gas used in this study contained about 99.998% N, while the air contained ~0.035% CO_2_, i.e., similar to atmospheric CO_2_ concentrations. The N_2_ purge likely removed not only O_2_ but also CO_2_ from the water. In contrast, dissolution of CO_2_ in the water of the systems purged with air probably increased the concentration of carbonic acid and led to a decrease in pH. In initially non-alkaline aqueous systems, pH tends to increase as a result of reduction reactions, as noted earlier in the discussion of the adsorption experiment. Still, sediment properties are likely to have played an important role in regulating the pH in the solution. For example, the pattern of pH changes under anoxic conditions differed in the Camp Creek and Roberts Creek systems *versus* the Gunder and till systems, especially on days 12 and 24. The Gunder sediments and the till were more alkaline than the Camp Creek and Roberts Creek sediments (the till was calcareous, with pH 8.1, Table 1), leading to a higher pH buffering capacity.

In all the systems, DOC was detected by day 12 of the experiment. Except for the Camp Creek system, anoxic conditions resulted in significantly higher DOC concentrations than did oxic conditions. However, even the highest mean DOC concentration in this study (1.4 mg L^-1^ for Camp Creek purged with N_2_) was about four-fold lower than the average DOC concentration of 6 mg L^-1^ reported by Schilling and Jacobson [22] in groundwater at a riparian well transect across Walnut Creek. Furthermore, DOC concentrations continued to increase on day 24 for the Camp Creek and Roberts Creek systems, while they tended to stabilize for the till and Gunder systems. This observation is probably linked to the level of organic matter in the sediment. Camp Creek and Roberts Creek sediments had organic matter contents of 3.38% and 4.51%, respectively; the values were higher than those in the Gunder sediments (1.51%) and the till (1.71%) (Table 1).

There were measurable increases in dissolved P concentrations in all the systems by day 6 (Fig 6). However, the release of P in this study was relatively slow compared to previous comparable lab experiments employing sediment and simulated stream water. For example, P release was recorded within the first day of the experiment conducted by Lai and Lam [49]. Similar results were reported by Kim *et al*. [50]. Further, Li *et al*. [51] found that P release occurred during the first 10 minutes. Differences in sediment types and methodology (e.g., solid-to-solution ratio, flow velocity, aerobic/anaerobic treatments) are likely to be the reasons for slower release of P in the present study compared to other studies. In addition, the use of dialysis tubing in this study separated the sediment and the water, whereas in the studies of Lai and Lam [49], Kim *et al*. [50], and Li *et al*. [51], there was direct contact between sediment and water. Thus, our reactor systems may have simulated the hyporheic zone more closely than previous studies.

Dissolved P concentrations released from all the sediments both under oxic and anoxic conditions increased from day 3 to day 12, then tended to be stable from day 12 to day 24. Li *et al*. [51] suggested that constant P concentrations in the water after a period of incubation indicated that an equilibrium state had been achieved. A similar mechanism is likely in the present study, where dissolved P concentrations remained stable starting on day 12. The stable dissolved P concentration varied among sediments and between the oxic and anoxic treatments, indicating that equilibrium P concentrations depend on the physicochemical properties of the sediment and water.

The highest dissolved P concentrations (~0.030 mg P L^-1^) were found on days 12 and 24 in anoxic conditions in the Camp Creek and Roberts Creek systems. These concentrations were two- to three-fold higher than the dissolved P concentrations in the oxic conditions in all sediments, as well as in anoxic conditions for the Gunder sediments and the till. The dissolved P concentrations measured in this experiment were comparable to those reported in a similar study by Jiang *et al*. [48].

Overall, the concentrations of dissolved P in the reactor experiment were within the range of measured dissolved P concentrations in four southern Iowa streams reported by Hongthanat et al. [44] and those reported for Walnut Creek in 2015 and 2016 by Shilling et al. [24]. Schilling *et al*. [24] also reported that dissolved P concentrations in groundwater sampled over that two-year period from wells in the Walnut Creek floodplain averaged 0.11 mg P L^-1^, but in groundwater sampled from upland wells averaged 0.05 mg P L^-1^. Schilling *et al*. [24] also found that dissolved oxygen concentrations (but not Eh values) were significantly lower in floodplain than in upland groundwater, and they suggested that a greater prevalence of anaerobic microsites in the floodplain sediments could have increased dissolved P concentrations in the groundwater.

After day 6, significant differences in dissolved P concentrations under oxic and anoxic conditions were found only in the Roberts Creek and Camp Creek sediment systems. The negative value of the mean differences shown in Table 3 indicates that dissolved P under anoxic conditions was higher than that under oxic conditions, which is comparable to the results reported by Lai and Lam [49], Kim *et al*. [50], and Li et al. [51]. In addition to the impact of decreasing Eh on the stability of P-binding Fe, Al, or Mn oxides, higher pH under anoxic conditions might have contributed to the higher dissolved P concentrations. As suggested by Jin et al. [52], higher pH values would increase the negative surface charge of Fe, Al, and Mn oxides, promoting the release of P from those solid phases.

The potential for carboxylic acid groups on dissolved organic matter to promote dissolution of P by anion exchange or by metal complexation was unclear in our study. Release of P from the sediments had begun by day 6 of incubation, but release of dissolved organic matter was not measured until day 12. Because the pH was higher under anoxic conditions, we expected DOC concentrations also to be higher. Under anoxic conditions, the solution concentration of DOC after 12 and 24 days was greater than under oxic conditions for Roberts Creek sediments, the Gunder sediments, and the till, but not for the Camp Creek sediments (Fig 6). It is possible that some organic matter was mobilized in colloidal forms but was unable to move through the dialysis tubing used to separate sediment solids from the liquid phase. Additional experiments addressing the potential for competition between dissolved organic matter and dissolved orthophosphate ions in these systems is warranted.

It is useful to note that, except for the incubated till systems, the equilibrium P values of the aerobic and anaerobic stirred reactor systems were generally lower than the corresponding EPC values for aerobic and anaerobic batch systems. For example, in the Camp Creek and Roberts Creek incubated systems, after 24 days of anaerobic incubation in the stirred reactors, the solution-phase P concentrations were much lower than the anaerobic EPC values for those sediments after 24 hours of incubation in the batch systems (Fig 6, Table 2). The 24-day P concentrations in both aerobic and anaerobic Gunder stirred reactor systems were about three times less than the EPC values in the aerobic batch systems and about eight times less than the EPC values in the anaerobic batch systems. For the till, the stirred reactor P concentrations were similar to the comparable EPC values. These observations highlight the impact on P release of physical disturbance to sediment particles and aggregates, and they suggest that model predictions of P sink–source relationships should rely on experimental systems that are as close as possible to the stream conditions of interest.

## Conclusions

The P sorption and release characteristics, represented by the equilibrium phosphorus concentration and phosphorus buffering capacity, varied among the bank sediments (Camp Creek, Roberts Creek, Gunder, and Pre-Illinoian Till) in the Walnut Creek watershed. When sediment samples were subjected to decreasing redox potential (Eh), there was a significant increase in EPC. Phosphorus desorption from the sediments was favored under low Eh. The batch experiment indicated that the EPC of the three Holocene-age sediments were similar to one another and increased with decreasing redox potential. In the stirred flow reactors, more dissolved P was released from the Camp Creek and Roberts Creek sediments under suboxic conditions than from the other sediments. This observation suggests that these two sediments, which are younger and higher in the stratigraphic sequence, could function as the primary internal P sources to Walnut Creek in anoxic settings, as in areas where groundwater is discharged as baseflow or in stagnant pools near debris dams. The P buffering capacity was greatest in the till. At locations in the channel where Pre-Illinoian till is in contact with the stream water, the till is likely to serve as an adsorbing sink for P dissolved in the water column.

Considered together, the results of this study highlight the differential impacts that sedimentary units can have on P mobilization in small watersheds. They also suggest that aqueous-phase equilibrium P concentrations (EPCs) that are based on samples shaken under aerobic conditions may not accurately predict P concentrations in water near stable sediments under oxic or suboxic conditions. Finally, models that incorporate variations in aqueous-phase redox potential are likely to improve predictions of P mobility in this and similar streams.
